# Release of volatile organic compounds (VOCs) from the lung cancer cell line CALU-1 *in vitro*

**DOI:** 10.1186/1475-2867-8-17

**Published:** 2008-11-24

**Authors:** Wojciech Filipiak, Andreas Sponring, Tomas Mikoviny, Clemens Ager, Jochen Schubert, Wolfram Miekisch, Anton Amann, Jakob Troppmair

**Affiliations:** 1 Department of Operative Medicine, Innsbruck Medical University Anichstr. 35, A-6020 Innsbruck, Austria; 2 Breath Research Unit of the Austrian Academy of Sciences, Innrain 66, A-6020 Innsbruck, Austria; 3 Institute for Ion Physics, Leopold-Franzens University of Innsbruck, A-6020 Innsbruck, Austria; 4 University of Rostock, Department of Anesthesiology and Intensive Care, Schillingallee 35, 18057 Rostock, Germany; 5 Daniel-Swarovski Research Laboratory, Department of Visceral, Transplant and Thoracic Surgery, Innsbruck Medical University, Innrain 66, A-6020 Innsbruck, Austria

## Abstract

**Background:**

The aim of this work was to confirm the existence of volatile organic compounds (VOCs) specifically released or consumed by lung cancer cells.

**Methods:**

50 million cells of the human non-small cell lung cancer (NSCLC) cell line CALU-1 were incubated in a sealed fermenter for 4 h or over night (18 hours). Then air samples from the headspace of the culture vessel were collected and preconcentrated by adsorption on solid sorbents with subsequent thermodesorption and analysis by means of gas chromatography mass spectrometry (GC-MS). Identification of altogether 60 compounds in GCMS measurement was done not only by spectral library match, but also by determination of retention times established with calibration mixtures of the respective pure compounds.

**Results:**

The results showed a significant increase in the concentrations of 2,3,3-trimethylpentane, 2,3,5-trimethylhexane, 2,4-dimethylheptane and 4-methyloctane in the headspace of CALU-1 cell culture as compared to medium controls after 18 h. Decreased concentrations after 18 h of incubation were found for acetaldehyde, 3-methylbutanal, butyl acetate, acetonitrile, acrolein, methacrolein, 2-methylpropanal, 2-butanone, 2-methoxy-2-methylpropane, 2-ethoxy-2-methylpropane, and hexanal.

**Conclusion:**

Our findings demonstrate that certain volatile compounds can be cancer-cell derived and thus indicative of the presence of a tumor, whereas other compounds are not released but seem to be consumed by CALU-1 cells.

## Background

Analysis of exhaled breath for recognition of human diseases offers the possibility of non-invasive diagnosis [1-4]. This is particularly interesting for critically ill persons [5], as well as for large scale screening, in the case of renal and liver diseases [6-10] or for cancer [11-17]. Exhaled air can be sampled as often as necessary without any restriction. It may even be done for newborn babies, or patients at the intensive care unit. Also *on-line *analysis of exhaled breath with continuous sampling and analysis of breath is possible [18-21].

A particularly ambitious goal is a better understanding of the biochemical background of endogenous compounds appearing in exhaled breath, both for healthy persons and persons suffering from certain diseases like cancer. Many compounds observed in breath have never been discussed in connection with physiological biochemical processes. Compounds like 2,2-diethyl-1,1-biphenyl or 2-methyl-1-(1,1-dimethylethyl)-2-methyl-1,3-propanediyl propanoic acid ester have been detected [11], which are potentially interesting but whose underlying biochemistry is completely unknown.

Better known volatile compounds in exhaled breath are methanol, ethanol, acetone, acetaldehyde and isoprene. Even for these compounds, a detailed *quantitative *understanding of production, metabolization and excretion is not easily available. *On-line *analysis of exhaled breath under a challenge (a test on an ergometer with varying pulse and heart rate, ingestion of food etc.). are promising and will provide information leading to a better quantitative understanding of biochemical processes within the human body.

In the present contribution, we turn towards compounds appearing in exhaled breath of cancer patients. Lung cancer patients present a changed pattern of concentration for many volatile compounds. Some compounds appear in increased concentration in exhaled breath, some of them are decreased in concentration [12-14]. For future cancer screening efforts it will be critical to know which of these compounds are effectively produced (or consumed) by cancer cells in the body. Other sources (and sinks) for volatile compounds are immuno-competent cells or microorganisms in the gut or the lung. These other sources are not considered here.

In the study presented here, we focus on a specific cancer cell line, CALU-1. In the future, we plan to extend our investigations to primary cells isolated by biopsies or in the course of a resection from patients themselves. This would allow a direct comparison of compounds released (or consumed) by cancer cells and the concentration patterns of volatile compounds in one and the same patient.

## Methods

### Cell Culture

As lung cancer cell line we tested the human, epithelial cell line CALU-1 which has been derived from a lung squamous cell carcinoma. This cell line builds numerous microvilli, a prominent rough endoplasmatic reticulum, lysosomes, and lipid inclusions. Furthermore, CALU-1 cells express a mutant K-ras gene. They are grown in DMEM high glucose (4.5 g/L) culture medium containing pyruvate (PAA) and supplemented with 10% FCS, penicillin (100 000 units/L), streptomycin (100 mg/L) and L-glutamine (293 mg/L). Cells were cultivated under standard conditions in a conventional incubator at 37°C in a humidified atmosphere with 92.5% air/7.5% CO_2_. For VOC measurements 50 millions trypsinized cells were inoculated in 100 ml phenol red free medium (DMEM high glucose) in a cell culture fermenter, flushed with clean, synthetic air taken from a gas cylinder (50 L defined gas mixture, Linde, Stadl-Paura, Austria) containing 5% CO_2 _for at least 10 min at a flow rate of 100 ml/min and sealed for 4 to 18 h. At the end of the incubation time 200 ml of air from the headspace was used for GC-MS analyses. Cell viability counts (trypan blue exclusion method) were performed at the end of the incubation period after.

### Sampling

Glass tubes (Gerstel, Mülheim an der Ruhr, Germany) filled with the following sorbents were used as traps for sample collection with simultaneous preconcentration: 25 mg Tenax TA (60/80 mesh), 35 mg Carboxen 569 (20/45 mesh), 250 mg Carboxen 1000 (80/100 mesh) (each from Supelco, Bellefonte, PA, USA). Sorbents were separated by glass wool. The sampled air was diluted 1:6 with dry and additionally purified synthetic air to avoid excess humidity for thermodesorption. The volume of collected sample originating from the fermenter was 200 ml with a total flow through sorption trap of 30 ml/min.

### Thermal desorption

The sampled analytes were released from sorbents by thermal desorption in the TDS3 unit equipped with a TDSA2 auto sampler (both from Gerstel, Mülheim an der Ruhr, Germany). The flow rate of carrier gas through the sorption trap during desorption was 90 ml/min. The initial temperature was 30°C and was increased to 300°C by a heating rate of 100°C/min (held for 10 min). Liquid nitrogen was used for cryofocusing the desorbed analytes (-90°C). For subsequent sample injection into the capillary column the CIS-4 injector which contained the glass liner filled with Carbotrap B (Gerstel, Mülheim an der Ruhr, Germany) was heated with the rate 12°C/sec up to 320°C (hold 2 min in splitless mode).

### GC-MS analyses

The TD-GC-MS analyses were performed on a 6890N gas chromatograph equipped with a mass selective detector 5973N (both from Agilent Technologies, Waldbronn, Germany) with sample injection by means of thermal desorption (described in previous sections). The MS analyses were performed in a full scan mode, with a scan range of 20–200 amu. Ionization of the separated compounds was done by electron impact ionization at 70eV. The acquisition of the chromatographic data was performed by means of the Agilent Chemstation Software (GC-MS Data Analysis from Agilent, Waldbronn, Germany) and the mass spectrum library NIST 2005 (Gatesburg, USA) was applied for identification. The PoraBond Q capillary column 25 m × 0,32 mm × 5 μm (Varian, Palo Alto, CA, USA) was used. The oven temperature program was as follows: initial 50°C held for 5 min, then ramped 5°C/min up to 140°C held 5 min, again ramped 5°C/min to 280°C and held for 4 min. The constant flow rate of helium as a carrier gas was 2 ml/min.

### Reagents and standards

2-Butanone, acrolein, methacrolein, 2-ethylacrolein, 2-methylpropanal, 3-methylbutanal, 2-methylbutanal, 2-methyl-2-butenal, hexanal, n-butyl acetate, methyl tert-butyl ether, ethyl tert-butyl ether, 2,4-dimethylpentane, 2,3,4-trimethylpentane, decane and tetrahydrofuran were each purchased from Sigma-Aldrich (Sigma-Aldrich, Steinheim, Germany). 2,3,3-Trimethylpentane, 2,3,5-trimethylhexane, 2,4-dimethylheptane and 4-methyloctane were purchased from ChemSampCo (ChemSampCo, LLC, Trenton, New Jersey, USA), acetaldehyde was purchased from Acros Organics (Acros Organics, Geel, Belgium) and acetonitrile was purchased from J.T. Baker (Mallinckrodt Baker B.V., Deventer, The Netherlands).

### Calibration

For quantification of compounds detected in headspace of cells and medium solutions the external standard calibration was performed. Preparation of gaseous standards was performed by evaporation of liquid substances in glass bulbs. Each bulb (Supelco, Bellefonte, PA, USA) was cleaned with methanol (Sigma-Aldrich, Steinheim, Germany), dried at 85°C for at least 20 hours, purged with clean nitrogen for at least 20 min and subsequently evacuated using a vacuum pump (Vacuubrand, Wertheim, Germany) for 30 minutes. Liquid standards (1–3 μL according to desired concentration) were injected through a septum, using a GC syringe. After the evaporation of standards the glass bulb was filled with nitrogen of purity 6.0 (i.e. 99,9999%, Linde, Vienna, Austria) in order to equalize the pressure (to the ambient pressure). Then the appropriate volume [μL] of vapour mixture was transferred using a gas tight syringe (Hamilton, Bonaduz, Switzerland) into Tedlar^® ^bags (SKC 232 Series, Eighty Four, PA, USA, SKC 232 Series) previously filled with 1,5 L of nitrogen (99,9999%) additionally purified by means of carbon molecular sieves (Carboxen 1000).

## Results

### Identification and quantification of VOCs released by CALU-1 cells

Viability after incubation of 50 millions of cells for 4 hours was 98.6 ± 1.1% and after incubation for 18 h 98.7 ± 0.7%. Thus, almost no cell death was caused under the given conditions which ensures that the release of potential VOCs is mostly due to living and not dying cells.

**Figure 1:**
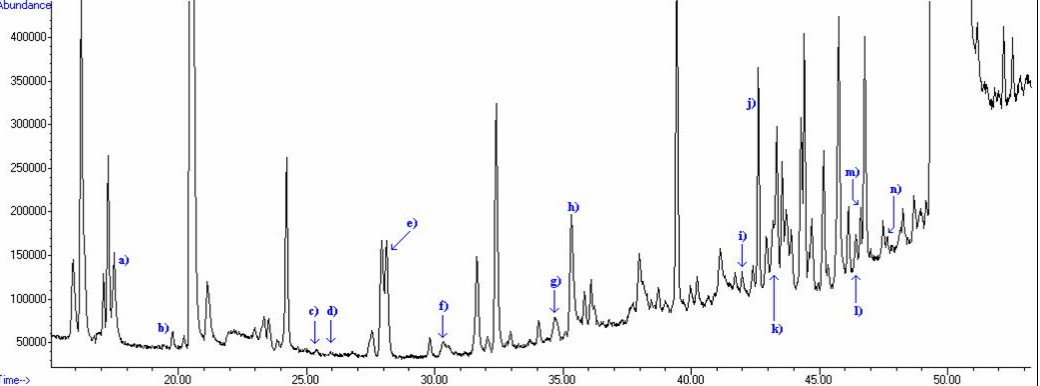
**An exemplary chromatogram of volatile compounds in headspace of the cancer cells CALU-1**. 200 ml of headspace from cells cultured in 100 ml medium were collected on a sorption trap filled with Tenax TA, Carboxen 569, Carboxen 1000 and subsequently thermal desorbed into the GC-MS system. The peaks that correspond to compounds discussed in this paper are labeled with letters (see Table 1). Peaks exceeding the range of scale are cut off.

Among all detected compounds we observed altogether 60 substances that could be identified not only by spectral library match using NIST 2005 library but also by determination of retention time based on calibration mixtures of the respective pure compound (Table 1A). An exemplary chromatogram of the headspace of CALU-1 cancer cells is presented in Figure 1. The substances, for which identification was done only by means of spectral library match without confirmation of their retention times, are listed in Table 1B. The peaks for which the proper identification was not possible (too low library match and no confirmation by retention time) are not discussed at all. Generally, the applied TD-GC-MS method is characterized by good linearity (even for the lowest detected concentrations) with correlation coefficients being mostly higher than 0,99. The LODs for almost all compounds were as low as ppt_v _level. The lowest LOD was observed for 2,3,3-trimethylpentane (127 ppt_v _= 5,725*10^-4 ^μg/l) and 2,3,5-trimethylhexane (137 ppt_v _= 7,583*10^-4 ^μg/l) but also polar compounds exhibited very low detection limits, e.g. 2-methylpropanal (143 ppt_v _= 3,912*10^-4 ^μg/l) and acetonitrile (147 ppt_v _= 1,166*10^-7 ^μg/l). Only two compounds of interest with LOD at the level of single ppb_v _(the highest observed values) were acetaldehyde (1,517 ppb_v _= 1,312*10^-3 ^μg/l), and 4-methyloctane (1,168 ppb_v _= 5,578*10^-3 ^μg/l). More detailed informations are listed in Tables 2 and 3A-B). Such low limits of detection with simultaneous low errors (expressed by correlation coefficients) testify very good precision and sensitivity of the applied TD-GC-MS method.

**Table 1:** 

**A: Summary of all substances identified by spectral match and confirmation of retention time**				
**No.**	**Compound**	**t_R _[min]**	**CAS**	
1	Methyl alcohol	8,778	67-56-1	
2	Dimethyl ether	10,323	115-10-6	
3	Acetaldehyde	11,936	75-07-0	
4	Methanethiol	13,130	74-93-1	
5	Isobutane	15,785	75-28-5	
6	2-Methyl-1-propene	16,118	115-11-7	
7	Ethyl alcohol	16,517	64-17-5	
8	Acetonitrile (a)	17,423	75-05-8	
9	2-Butene (Z or E isomer)	17,009	107-01-7	
10	Butane	17,186	106-97-8	
11	2-Butene (E or Z isomer)	17,360	115-11-7	
12	2-Propenal (b)	19,310	107-02-8	
13	Furan	19,741	110-00-9	
14	Propanal	20,170	123-38-6	
15	Acetone	20,469	67-64-1	
16	Isopropyl Alcohol	21,720	67-63-0	
17	2-Methylbutane	23,229	78-78-4	
18	Ethyl ether	23,503	60-29-7	
19	1-Propanol	23,696	71-23-8	
20	Pentane	24,224	109-66-0	
21	Methacrolein (c)	25,375	78-85-3	
22	2-Methylpropanal (d)	25,917	78-84-2	
23	Methyl vinyl ketone	26,795	78-94-4	
24	Trichloromethane	27,561	67-66-3	
25	Tetrahydrofuran	27,920	109-99-9	
26	2-Butanone (e)	28,124	78-93-3	
27	Ethyl acetate	29,829	141-78-6	
28	2-Methoxy-2-methylpropane (f)	30,377	1634-04-4	
29	2-Methyl-1-pentene	31,692	763-29-1	
30	Benzene	32,418	71-43-2	
31	Hexane	33,003	110-54-3	
32	2-Ethylacrolein	34,049	922-63-4	
33	3-Methylbutanal (g)	34,587	590-86-3	
34	2-Ethoxy-2-methylpropane (h)	35,346	637-92-3	
35	2-Pentanone	35,868	107-87-9	
36	Methyl methacrylate	36,121	80-62-6	
37	2-Methyl-2-butenal	37,277	1115-11-3	
38	3-Methylhexane	38,434	589-34-4	
39	Cyclopentanone	38,750	120-92-3	
40	Toluene	39,466	108-88-3	
41	Furfural	40,044	98-01-1	
42	Methyl isobutyl ketone	40,262	108-10-1	
43	Hexanal (i)	42,001	66-25-1	
44	Butyl acetate (j)	42,624	123-86-4	
45	2,3,4-Trimethylpentane	42,949	565-75-3	
46	2,3,3-Trimethylpentane (k)	43,202	560-21-4	
47	4-Methylheptane	43,385	589-53-7	
48	3-Ethylhexane	43,580	619-99-8	
49	Octane	44,465	111-65-9	
50	p-Xylene	44,721	106-42-3	
51	Styrene	45,195	100-42-5	
52	p-Xylene	45,363	106-42-3	
53	3-Methyl-2(5H)-furanone	45,779	22122-36-7	
54	2,3,5-Trimethylhexane (l)	46,456	1069-53-0	
55	2,4-Dimethylheptane (m)	46,647	2213-23-2	
56	Benzaldehyde	46,799	100-52-7	
57	4-Methyloctane (n)	47,697	2216-34-4	
58	Octanal	50,921	124-13-0	
59	Limonene	52,217	138-86-3	
60	Decane	52,572	124-18-5	

Listed are substances observed in the headspace of medium as well as in the headspace of CALU-1 cancer cells. Letters in parentheses refer to peaks shown in the chromatogram of Figure 1.				

**B: List of compounds identified only by means of spectral library match**				
	**Compound**	**t_R _[min]**	**CAS**	**M**^+.^

	Propene	7,939	115-07-1	42
	Chloromethane	8,259	74-87-3	50
	Acetophenone	51,165	98-86-2	120
	(1E)-1-Butenylbenzene	53,069	824-90-8	132
	Nonanal	54,630	124-19-6	144

Listed are compounds detected in the headspace of medium and CALU-1 cancer cells. Relative molecular mass (RMM) of the suspected molecular ion is given for each presented compound.				

**Table 2:** Quantification of VOCs released from CALU-1 cancer cells after adsorption on solid sorbents with subsequent TD-GC-MS analyses.

				**Concentration [ppb_v_] after 18 hours of incubation**						**mean ppb Medium**	**mean ppb Cells**	**ppb Cells/ppb Medium**
**Compound**	**CAS**	**R^2^**	**LOD [ppb_v_]**	**Medium**			**Cells**					

**2,3,3-trimethylpentane**	560-21-4	0,998	0,127	0,37	0,45	0,40	0,60	0,84	0,86	0,41	0,77	1,89
**2,3,5-trimethylhexane**	1069-53-0	0,997	0,149	0,43	0,42	0,29	0,79	0,67	0,50	0,38	0,65	1,71
**2,4-dimethylheptane**	2213-23-2	0,996	0,179	0,33	0,35	0,20	1,25	0,77	0,65	0,29	0,89	3,04
**4-methyloctane**	2216-34-4	0,985	1,168	1,33	1,51	2,02	4,03	2,09	2,17	1,62	2,76	1,71

The concentrations of these VOCs are increased in headspace of CALU-1 cells in comparison with medium only, after 18 hours of incubation. The concentrations of discussed VOCs are given for each measured sample (headspace of CALU-1 cells and of medium). The correlation coefficients (for calibration experiments) and the limits of detection (LODs) are presented.

### TD-GC-MS analyses of the headspace of CALU-1 cells

In three independent experiments using TD-GC-MS after preconcentration of sampled air by means of adsorption on solid sorbents, the concentrations of 4 compounds could be shown to be increased (Table 2) and other 12 compounds to be decreased (Table 3A and 3B) in the headspace of CALU-1 cells as compared to the headspace of medium only. These compounds are those which were detected in all measured samples of cancer cells as well as medium. All other 44 (= 60-16) identified compounds are not discussed in detail since they appeared "irregularly", i.e., not consistently increased or decreased with respect to medium only. The only exception is 2-methyl-2-butenal which was always found > LOD in cell culture medium samples whereas its concentration was below LOD in *every *CALU1 cell sample. Because of the big variations and small sample population, statistical tests should not be used. Instead, the direct comparison of VOCs concentration detected in cell and medium samples was done. We discuss only those compounds which are present in *every *CALU1 cell sample at (consistently) higher or (consistently) lower level than in *every *medium sample. Ratios of mean concentrations from both kinds of samples are given in Tables 2 and 3A–B.

**Table 3:** A and B: Quantification of VOCs consumed by CALU-1 cancer cells after adsorption on solid sorbents with subsequent TD-GC-MS analyses.

**A**														
				**Concentration [ppb_v_] after 4 hours of incubation**								**mean ppb Medium**	**mean ppb Cells**	**ppb Cells/ppb Medium**
**Compound**	**CAS**	**R^2^**	**LOD [ppb_v_]**	**Medium**				**Cells**						
**methacrolein**	78-85-3	0,999	0,466	5,66	9,38	3,93	0,63	0,34	0,84	0,34	0,34	4,898	0,469	0,10
**2-methylpropanal**	78-84-2	0,998	0,143	19,80	12,98	39,53	9,12	1,21	below LOD	3,35	2,03	20,357	1,649	0,08
**methyl tert-butyl ether**	1634-04-4	0,999	0,293	1,68	3,43	3,56	1,60	0,67	0,94	1,30	1,11	2,567	1,004	0,39
**3-methylbutanal**	590-86-3	0,994	0,234	142,29	184,19	218,74	73,14	0,08	2,44	3,02	2,13	154,588	1,916	0,01
**n-butyl acetate**	123-86-4	0,999	0,134	60,47	105,13	66,58	58,08	2,01	14,70	11,27	13,08	72,566	10,267	0,14
														
**B**														
**Compound**	**CAS**	**R^2^**	**LOD [ppb_v_]**	**Concentration [ppb_v_] after 18 hours of incubation**								**mean ppb Medium**	**mean ppb Cells**	**ppb Cells/ppb Medium**
					**Medium**			**Cells**						

**acetaldehyde**	75-07-0	0,993	1,517	257,527	335,818	315,063		below LOD	116,472	81,692		302,803	66,054	0,22
**acetonitrile**	75-05-8	0,998	0,147	11,344	7,540	5,764		4,183	3,633	4,100		8,216	3,972	0,48
**acrolein**	107-02-8	0,998	0,718	5,703	3,462	4,079		2,185	2,071	3,238		4,415	2,498	0,57
**methacrolein**	78-85-3	0,999	0,466	5,933	4,975	4,723		0,811	0,344	0,702		5,210	0,619	0,12
**2-methylpropanal**	78-84-2	0,998	0,143	30,915	11,773	21,277		0,532	3,573	0,083		21,322	1,396	0,07
**2-butanone**	78-93-3	0,999	0,149	3,523	5,206	3,084		1,114	2,812	1,669		3,938	1,865	0,47
**methyl tert-butyl ether**	1634-04-4	0,999	0,293	2,126	4,202	2,089		0,753	1,654	1,023		2,806	1,143	0,41
**3-methylbutanal**	590-86-3	0,994	0,234	157,462	206,895	78,027		2,226	2,215	1,529		147,461	1,990	0,01
**ethyl tert-butyl ether**	637-92-3	0,999	0,215	6,168	11,281	5,121		2,060	4,805	2,229		7,523	3,032	0,40
**2-methyl-2-butenal**	1115-11-3	0,985	0,430	1,839	1,024	1,163		below LOD	below LOD	below LOD		1,342	0,000	0,00
**hexanal**	66-25-1	0,981	0,540	2,043	2,805	1,980		0,791	1,016	0,711		2,276	0,839	0,37
**n-butyl acetate**	123-86-4	0,999	0,134	43,949	31,381	26,348		11,084	3,032	3,324		33,892	5,813	0,17

The concentration of these VOCs is decreased in headspace of CALU-1 cells in comparison with medium only. Two different incubation times were studied: 4 h (A) and 18 h (B). The concentrations of discussed VOCs are given for measured sample (headspace of CALU-1 cells and of medium). The correlation coefficients (for calibration experiments) and the limits of detection (LODs) are presented.

**Figure 2:**
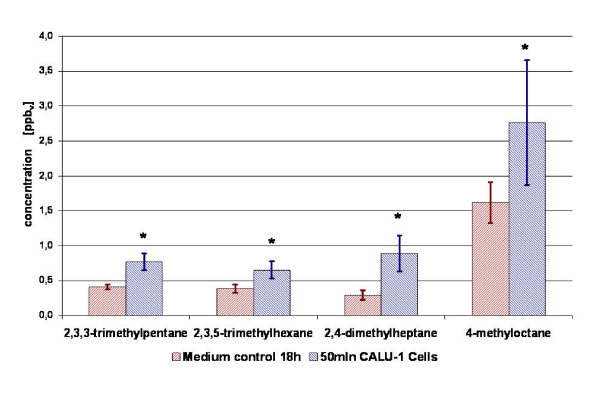
**Comparison of VOCs concentrations present at higher concentration in the headspace of CALU-1 cells in culture (blue columns) as compared to medium control (red columns) after 18 h of incubation**. Presented are average concentrations (n = 3) with standard deviations. Significant differences are labeled with asterisks.

**Figure 3:**
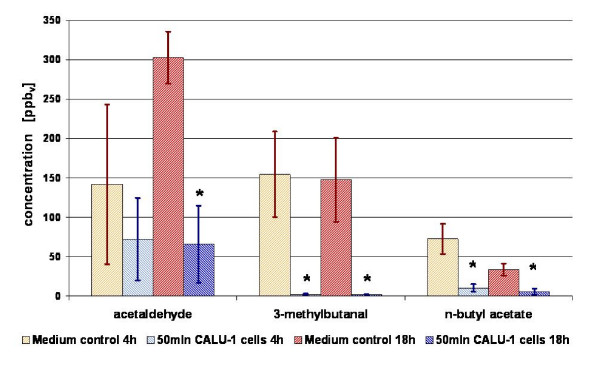
**Comparison of VOCs present at lower concentrations present in the headspace of cells in culture (CALU-1) (bright blue and dark blue columns) as compared to medium control (yellow and red columns) after 4 h and 18 h of incubation**. Presented are average concentrations (n = 4 for 4 h of incubation, n = 3 for 18 h of incubation) with standard deviations. Significant differences are labeled with asterisks.

**Figure 4:**
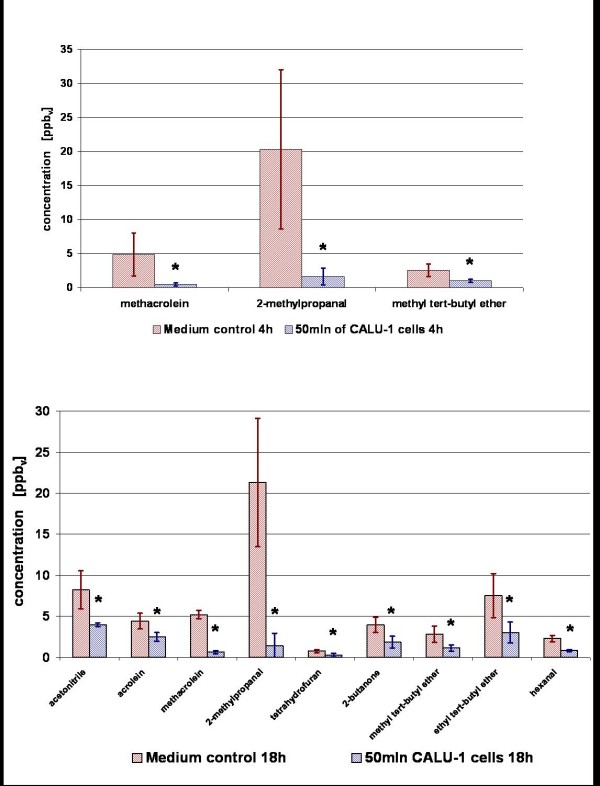
**A and B**: **Comparison of VOCs concentrations present at lower level in the headspace of cell culture (CALU-1) solution (blue columns) as compared with medium only (red columns) after 4 h (Fig. 4A) and 18 h (Fig. 4B) of incubation.** Presented are average concentrations (n = 4 for 4 h of incubation, n = 3 for 18 h of incubation) with standard deviation. Significant differences are labeled with asterisks.

After 4 hours of incubation in the fermenter an increase of isopropyl alcohol could be found. However, concentration of 2-propanol increased in only 3 from 4 measurements with 4 h incubation, whereas for the samples incubated 18 h there is no significant difference between the measured amount of 2-propanol in headspace of cancer cells and in headspace of medium. After 18 h of incubation 2,3,3-trimethylpentane (mean ppb_v _in cells/mean ppb_v _in medium = 1,89), 2,3,5-trimethylhexane (1,71), 2,4-dimethylheptane (3,04) and 4-methyloctane (1,71) were *increased *significantly in the headspace of CALU-1 cells (Figure 2). Decreased concentrations from cancer cells after 4 h were found for the compounds methacrolein (0,10), 2-methylpropanal (0,08), 2-methoxy-2-methylpropane (0,39), 3-methylbutanal (0,01), 2-methyl-2-butenal (0) and n-butyl acetate (0,14) (Figure 3 and Figure 4A). After 18 h of incubation acetaldehyde (mean ppb_v _in cells/mean ppb_v _in medium = 0,22), acetonitrile (0,48), acrolein (0,57), methacrolein (0,12), 2-methylpropanal (0,07), 2-butanone (0,47), 2-methoxy-2-methylpropane (0,41), 3-methylbutanal (0,01), 2-methyl-2-butenal (0), 2-ethoxy-2-methylpropane (0,40), hexanal (0,37) and n-butyl acetate (0,17) were decreased significantly (Figure 3 and Figure 4B).

## Discussion

In our experiments VOCs released from the lung cancer cell line CALU-1 were collected and preconcentrated by means of adsorption on solid sorbents with subsequent thermal desorption and analysis by GC-MS. In investigations with the cell line CALU-1 longer and shorter incubation times (18 h and 4 h respectively) were tested whereas in earlier experiments a technical device and a protocol for this experimental setting had to be developed.

The results of experiments with CALU-1 cells confirmed the existence of compounds that are either released or consumed by these cells. Discussed are only these analytes which were detected in all samples of medium as well as cancer cells. Furthermore, it is indicated that the release of compounds can be detected after prolonged incubation time but so far no significant release after 4 hours of incubation could be observed. On the other hand, consumption of several aldehydes as well as n-butyl acetate and methyl tert-butyl ether from the headspace of cells solution could be detected already after a short period of incubation. Even for the compounds like acetaldehyde and especially 3-methylbutanal and n-butyl acetate that are based on high background from medium, a strong reduction of concentration was observed after only 4 hours of incubation (Table 3A). The influence of incubation time on the amount of detected compounds is illustrated in Figure 3. Because of big variations in measured peak areas of acetaldehyde the significance for this compound was not confirmed after 4 h incubation, although a tendency can be observed.

Interestingly, the results of our experiments showed that the majority of detected volatile substances which are significantly increased in the headspace of cell culture compared to medium control are saturated hydrocarbons. From CALU-1 cells 2,3,3-trimethylpentane, 2,3,5-trimethylhexane, 2,4-dimethylheptane and 4-methyloctane were released (Figure 2). The measured concentrations of these compounds are listed in Table 2. In the group of Phillips et al. it was found that several alkanes and branched hydrocarbons including also 4-methyloctane were increased in the breath of lung cancer patients compared to healthy persons [12,13].

Because of the high concentrations of target compounds in the medium, the difference between their amount in cell and in medium headspace samples is rather low. Moreover, although release of some compounds seems to be observed, background peak areas are considerably different in independent experiments which results in high standard deviation. Thus direct comparison of measured amounts is not precise. The compounds already present in the headspace of culture medium may, in particular, originate from fetal calf serum added to culture medium. Thus, to reduce background one option would be to use medium without serum or to reduce serum to low concentrations. However, preliminary experiments indicated a loss of VOC release under low serum cell culture conditions, possibly due to different metabolism of the cell line used. Thus to achieve lower background level we reduced fetal calf serum but only to 5%.

The metabolic origin of the detected substances so far remains speculative. In the present experiments we observed saturated as well as branched hydrocarbons, which corroborates clinical studies which demonstrate exhalation of hydrocarbons.

We observed that acetaldehyde is consumed by CALU-1 cells (Figure 3). This fact is contradictory to the work of Smith et al. who found increased concentrations of acetaldehyde released from CALU-1 and SK-MES cells, also a lung cancer cell line [22]. The reason or reasons for this completely opposite behavior of CALU-1 cells, concerning the metabolism of acetaldehyde are unclear. In literature acetaldehyde is described as the first metabolic product of alcohol metabolism *in vivo *[23] and as carcinogenic.

Besides acetaldehyde several other compounds are consumed or taken up by the cells whereby especially aldehydes have decreased concentrations compared to medium control (see Table 3A and 3B and Figures 4A and 4B). As for acetaldehyde the mechanism of consumption of those substances by the cells has to be elucidated. However, the degradation or consumption of aldehydes may be partly explained by an increased activity of aldehyde dehydrogenases. For instance, aldehyde dehydrogenase 1 was found to be a marker of breast cancer cells [24] and was found to be increased in the lung cancer cell line A549 [25]. Moreover, in a work of Patel et al. [26] significantly higher expression levels of aldehyde dehydrogenase 1A1 and aldehyde dehydrogenase 3A1 were detected in squamous cell cancer, adenocarcinoma, and small cell lung cancer. In this work it was shown that atypical pneumocytes demonstrated significantly higher levels of expression of aldehyde dehydrogenase 1A1 and aldehyde dehydrogenase 3A1 than normal pneumocytes (a normal counterpart of adenocarcinoma), which is suggestive of up regulation during malignant transformation to adenocarcinoma. It was also found that non-small cell lung cancer expresses very high levels of aldehyde dehydrogenase 1A1 and aldehyde dehydrogenase 3A1 in comparison with small cell lung cancer. Thus, elevated expression of both enzymes may be associated with malignant transformation to adenocarcinoma. Those data would point out a putative metabolic mechanism of degradation of aldehydes which was observed in this work.

## Conclusion

This work is a first step to get a better understanding on the metabolism of VOCs from cancer cells. We found that 2,3,3-trimethylpentane, 2,3,5-trimethylhexane, 2,4-dimethylheptane and 4-methyloctane are released from the cell line CALU-1 and therefore should be taken into account as possible tumor-related compounds. It was also clearly shown that these cells are consuming several types of aldehydes but also compounds from other classes. Decreased level of aldehydes can be partly explained by higher activity of aldehyde dehydrogenase in cancer cells. Summarizing, these investigations give some hints which substances are candidates for biomarkers of cancer disease and thus may finally provide a technically feasible method for early and non-invasive diagnosis of lung cancer. However, biochemical background of all discussed compounds should be elucidated before using them as fully assured biomarkers.

## Competing interests

The authors declare that they have no competing interests.

## Authors' contributions

The original plan of the cell culture workpackage in the EU-project BAMOD was devised and written by JS, WW, AA and JT. WF performed the gas chromatographic analysis of all samples, performed the calibrations and wrote a draft of the manuscript. AS performed the cell culture experiments and wrote a draft of the manuscript. TM developed the cell culture measuring device. CA did the data analysis. JS, WF and WM developed the GCMS-measurement protocol for measurement of volatile compounds in headspace of cell cultures (choice of column and GCMS temperature protocol). AA and TJ designed the study, supervised the experiments, discussed the results and continuous improvement of measurements using different analytical techniques, and finalized the manuscript. All authors read and approved the manuscript.
